# Society Meetings

**Published:** 1899-05

**Authors:** 


### SOCIETY MEETINGS.

THE Medical and Chirurgical Faculty of Maryland held its centennial meeting in McCoy Hall, at Johns Hopkins University, April 25-28, 1899, under the presidency of Dr. S. C. Chew, of Baltimore.

Several public receptions were held. Dr. W. W. Keen, of Philadelphia, delivered the annual oration, and among other papers read was one by Dr. Roswell Park, of Buffalo, entitled, Cancer as a parasitic disease. Drs. Jacobi, of New York; George Ben Johnston, of Richmond, and other invited guests also read papers. There were a number of private receptions given, a luncheon was held at Johns' Hopkins Hospital University, and the annual dinner was attended on Thursday evening, April 27th. The Medical and Chirurgical Faculty of Maryland corresponds to the state medical societies in other commonwealths, and it is to be congratulated upon its most successful centennial celebration.

---

THE section on materia medica, pharmacy and therapeutics of the American Association urges those who desire to read papers in its departments at the Columbus meeting, June 6th to 9th, to send their names and the titles of their papers at once to the secretary, Dr. Leon L. Solomon, secretary, 323 W. Walnut street, Louisville, Ky., who is now making up the final program.

---

THE third district branch of the New York State Medical Association will hold its next meeting at Elmira, N. Y., June 1, 1899, under the presidency of Dr. F. D. Reese. Several members of the association from other parts of New York State, including the president of the State association, Dr. Joseph D. Bryant, are expected to be present.

Titles of papers to be presented at this meeting should be sent to the acting secretary, Dr. F. W. Higgins, of Cortland, without delay.

---

THE American Medical Editors' Association and the American Medical Publishers's Association propose holding a joint session at Columbus, O., previous to or during the meeting of the American Medical Association, which latter has been fixed for June 6, 1899.

---

THE Third International Congress for Gynecology and Obstetrics will take place at Amsterdam, August 8-12, 1899, under the patronage of the Minister of the Interior. The leading questions for discussion will be the following:

(1) The surgical treatment of fibro-myoma. (2) The relative value of antisepsis and improved technic for the actual results in gynecological surgery. (3) The influence of posture on the form and dimensions of the pelvis. (4) The indication for Cesarean section compared to that for symphyseotomy, craniotomy and premature induction of labor.

The committee has obtained the concurrence as reporters of M. M. Doyen, Howard Kelly and Schauta, who will treat the first question; M.M. Bumm, Richelot and Lawson Tait the second; M.M. Bonnaire, Pinzani and Walcher the third, and M.M. Leopold, Pinard, Pestalozza and Fancourt Barnes the fourth. As regards private communications, preference will be given to those bearing upon the above mentioned leading questions. Time will also be allowed sufficient for any demonstrations kindly afforded by the members. The official languages are: English, French, German and Italian.

The committee is made up as follows: H. Treub, president; J. Veit, vice-president; G. C. Nijhoff, J. P. Barnouw, treasurer; M. A. Mendes de Leon, secretary. The address of the secretary is Sarphatistraat 1H., Amsterdam.

---

THE American Association of Obstetricians and Gynecologists will hold its twelfth annual meeting at Indianapolis, Tuesday, Wednesday and Thursday, September 19, 20 and 21, 1899, under the presidency of Dr. Edward J. Ill, of Newark, N. J, Dr. Ill has appointed the following-named delegates to represent the association at the Third International Congress of Gynecology and Obstetrics, to be held at Amsterdam, August 8-12, 1899: Dr. J. H. Carstens, of Detroit; Dr. Clinton Cushing, of Washington; Dr. W. E. B. Davis, of Birmingham; Dr. B. Sherwood-Dunn, of Boston; Dr. L. H. Dunning, of Indianapolis; Dr. George Ben Johnston, of Richmond; Dr. L. S. McMurtry, of Louisville; Dr. J. B. Murphy, of Chicago; Dr. Charles A. L. Reed, of Cincinnati; Dr. A. Vander Veer, of Albany and Dr. X. O. Werder, of Pittsburg. The State Department at Washington has also issued credentials to these delegates, duly accrediting them by the signature of the Secretary of State and the seal of the department as representatives of the government at the congress.

---

THE Mississippi Valley Medical Association has changed the date of its annual meeting from September 12-15 to October 3-6, 1899, inclusive.

The autumn fete to be known as the American festival, will be held in Chicago, beginning September 25th and ending October 9th with the laying of the corner stone of the Federal building when the

President and the Cabinet will be in the city. During this time the railroad fare to Chicago from all points will be a flat one fare rate for the round trip, without the necessity of certificates or signatures. The limit of the tickets is so long that a protracted stay can be made in Chicago in order to take advantage of the clinical facilities of the meeting as well as enjoy the added attractions of the festival.

---

THE Buffalo Academy of Medicine held meetings during the months of March and April, 1899, as follows:

*Section on Obstetrics.*—Tuesday evening, March 28th, program: Hemorrhage after labor, Herman E. Hayd.

*Section on Surgery.*—Tuesday evening, April 4th, program: Acute osteomyelitis, Roswell Park. Discussion by Herman Mynter, Hartwig and others. Report of case, Chauncey Pelton Smith.

*Section on Medicine.*—Tuesday evening, April 11th, program: Hereditary syphilis, A. E. Diehl. Nasal and cranial diseases, Wm. C. Krauss.

*Section on Pathology.*—Tuesday evening, April 18th, program: All members were invited to join in a discussion of the relations between renal elimination and general disease. The discussion was opened by Drs. Thomas B. Carpenter, Henry Ingraham and Edmond E. Blaauw.

*Section on Obstetrics.*—No meeting was held Tuesday evening, April 25, 1899, on account of the commencement exercises of the University of Buffalo.

---

THE Niagara Falls Academy of Medicine held its regular meeting at the office of Dr. Guillemont, Monday evening, May 1, 1899, at which time Dr. F. T. Jenkins read a paper. At the April meeting the following nominations were made for officers for the ensuing year: President, Drs. Scott, McBrien and LeoWolf; vice-president, Drs. Guillemont and Falkner; secretary, Drs. LeoWolf, Scott and McCarty; treasurer, Drs. LeoWolf, Scott and McCarty; trustees, Drs. Bingham and Hall.

---

A CONGRESS for the study of tuberculosis will be held in Berlin from May 24 to 27, 1899. This congress, under the protectorate of the Empress of Germany, will go into a thorough study of the extent, etiology, prophylaxis, therapy and segregation of tuberculosis and promise results of greatest value and import. The meetings are to be held in the Reichstag building, under the presidency of Prince Hohenlohe, the chancellor, and Professor E. von Leyden and Dr. Pannwitz, secretary. Two meetings will be held each day, at 9 a. m. and 2 p. m. The bureau of the organisation will be at Wilhelm Platz, 2 Berlin W.

---

Rectal Specialists.—At the time of the meeting of the American Medical Association at Columbus, June 6-9, there will be a meeting of the medical men engaged in the practice of proctology, for the purpose of effecting a permanent society for the study of their specialty. Physicans interested in the project are requested to address Dr. Wm. M. Beach, 515 Penn avenue, Pittsburg, Pa.

---

The American Climatological Association will hold its sixteenth annual meeting at the Academy of Medicine, New York City, May 9, 10 and 11, 1899, under the presidency of Dr. Beverly Robinson, of New York. An exceedingly interesting and elaborate program has been prepared and pleasant social entertainments have been provided. These latter are as follows: The members are invited to luncheon daily at the close of the morning session at the Academy of Medicine; Mrs. Beverly Robinson will receive wives of the members at her residence, 42 West Thiity-seventh street, on May 9th, from 4 to 6 o'clock; a smoker will be tendered the association at Delmonico's on the evening of the first day, May 9th, from 9 to 12 p. m., and the annual dinner will be served on the evening of the second day, May 10th, at 7 o'clock, at the Hotel Manhattan. An excursion to the Loomis Sanitarium, at Liberty, N. Y., has been arranged for May 11th, the third day of the meeting. Members of the profession are cordially invited to attend the meetings of the association. Dr. Guy Hinsdale, of Philadelphia, is the secretary.

